# Treatment of esophageal tumors using high intensity intraluminal ultrasound: first clinical results

**DOI:** 10.1186/1479-5876-6-28

**Published:** 2008-06-05

**Authors:** David Melodelima, Frederic Prat, Jacques Fritsch, Yves Theillere, Dominique Cathignol

**Affiliations:** 1 Inserm, U556, Lyon, F-69003, France; 2 Université de Lyon, Lyon, F-69003, France; 3 Department of gastroenterology and digestive cancers, Cochin Hospital, F-75014 Paris, France; 4 Department of endoscopy, Centre Hospitalier Bicetre, F-94275 Le Kremlin Bicetre, France

## Abstract

**Background:**

Esophageal tumors generally bear a poor prognosis. Radical surgery is generally the only curative method available but is not feasible in the majority of patients; palliative therapy with stent placement is generally performed. It has been demonstrated that High Intensity Ultrasound can induce rapid, complete and well-defined coagulation necrosis. Thus, for the treatment of esophageal tumors, we have designed an ultrasound applicator that uses an intraluminal approach to fill up this therapeutic gap.

**Methods:**

Thermal ablation is performed with water-cooled ultrasound transducers operating at a frequency of 10 MHz. Single lesions extend from the transducer surface up to 10 mm in depth when applying an intensity of 14 W/cm^2 ^for 10s. A lumen inside the therapy applicator provides path for an endoscopic ultrasound imaging probe operating at a frequency of 12 MHz. The mechanical rotation of the applicator around its axis enables treatment of sectorial or cylindrical volumes. This method is thus particularly suitable for esophageal tumors that may develop only on a portion of the esophageal circumference. Previous experiments were conducted from bench to *in vivo *studies on pig esophagi.

**Results:**

Here we report clinical results obtained on four patients included in a pilot study. The treatment of esophageal tumors was performed under fluoroscopic guidance and ultrasound imaging. Objective tumor response was obtained in all cases and a complete necrosis of a tumor was obtained in one case. All patients recovered uneventfully and dysphagia improved significantly within 15 days, allowing for resuming a solid diet in three cases.

**Conclusion:**

This clinical work demonstrated the efficacy of intraluminal high intensity ultrasound therapy for local tumor destruction in the esophagus.

## Background

Most occurrences of esophageal tumors are not amenable to curative resection because of the extent of the tumor at diagnosis and the presence of a comorbid condition. Chemotherapy and/or radiochemotherapy have not demonstrated any survival advantage so far. In this context, new methods for local tumor destruction, such as the use of physical agents, have raised interest. For example, endoscopic clearance of the esophagus is already routine with laser photocoagulation [1,2] being widespread although its action remains superficial and several sessions are generally needed to achieve symptomatic improvement. Indeed esophageal tumors, which develop inside the circumference of the lumen, are very difficult to treat by physical agents for two reasons. They are sometimes sector-based and the tumor thickness can vary significantly from one point to another (three millimeters to 12 mm in most cases). Thus, for this form of cancer, a minimally invasive local treatment, leading to an immediate, deep and complete destruction of the targeted tissues, could be curative in the earliest-detected cases. If the tumor has reached a metastatic stage, the treatment could be envisaged as a palliative strategy to delay the use of stents and increase the median survival time. Among the heating modalities for therapeutic thermal ablation, tissue coagulation by high intensity ultrasound is a well-established method of tumor treatment [3-6]. Treatments by ultrasound are well-delimited; damages to surrounding tissues are minimal. Excellent results have been obtained (both experimentally and clinically) in inducing homogeneous and reproducible tumor destruction by thermal coagulation necrosis. Recently, several authors have suggested using miniaturized applicators for therapeutic ultrasound [7-9], which makes it an approach that is particularly appropriate for carcinoma of the esophageal ducts.

In this study, a plane transducer was used to generate single parallelepiped-shaped lesions in front of the emitting face. Since the ultrasonic beam does not diverge in the near field, the pressure drop along the ultrasonic axis is only dependent on the energy attenuation in tissues[10]. Each single lesion can be induced in 10 seconds to a depth up to 16 mm, and with almost no perfusion-dependence [11]. Furthermore, by rotating the applicator around its axis after each ultrasound exposure, a sector-based or cylindrical volume can be coagulated. *Ex vivo *experiments conducted on pig liver demonstrated the ability of the applicator to generate any angular sector-based lesion with a good angular accuracy by rotating the transducer and juxtaposing single lesions. The dimensions of the cylindrical lesions were associated with the exposure conditions. In addition, preliminary experiments conducted *in vivo *on pig esophagi have shown the feasibility of the treatment [11,12] and allowed the determination of the thermal dose that can be administered to different regions of the esophagus. In this paper we describe an open pilot trial which was conducted between Sept, 2002 and Nov, 2004 on four patients with adenocarcinoma or squamous cell carcinoma. The aim of this study was to investigate the safety and feasibility of intraluminal high intensity ultrasound for the treatment of esophageal tumors.

## Methods

### Ultrasound equipment

**Figure 1:**
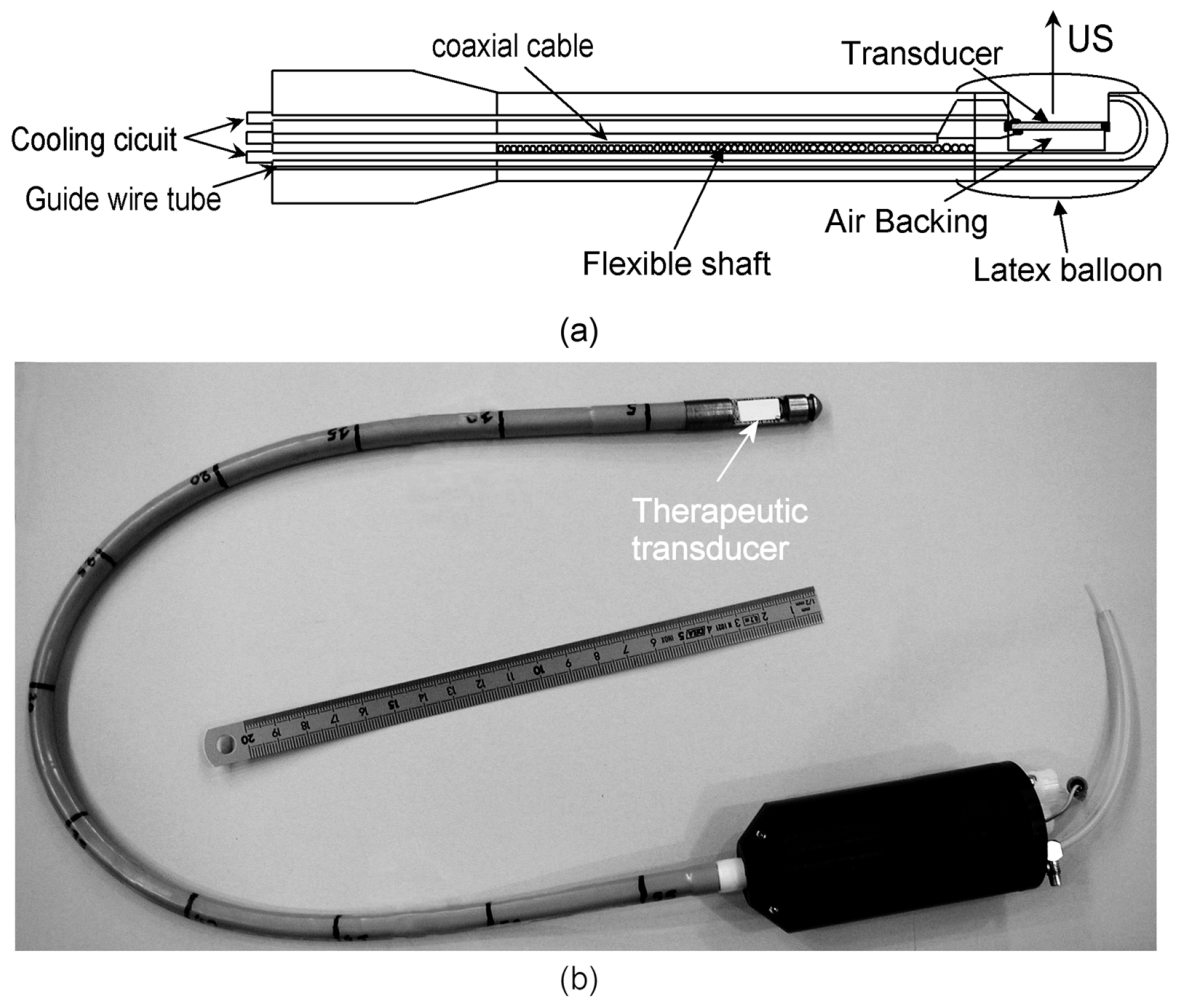
**Intraluminal ultrasound applicator**. (a) Schematic diagram of the applicator. (b) US device for the treatment of esophageal tumors.

**Figure 2:**
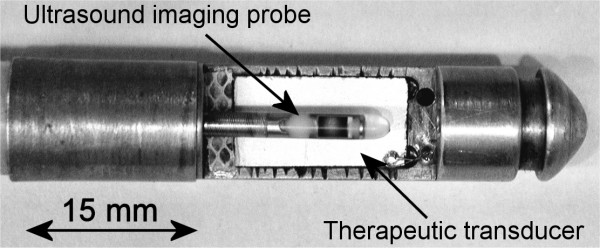
**Head of the intraluminal high intensity ultrasound applicator**. The ultrasound imaging probe is in front of the therapeutic transducer to image the region of interest.

The head of the applicator is made of brass and round-shaped to permit transesophageal application without risk of injury (Fig. 1). The ultrasound emitter is a 15 × 8 mm^2 ^piezoceramic plane transducer (Quartz & Silice, P7-62, Nemours, France) which operates at 10.4 MHz. This air-backed transducer was sealed with epoxy resin (Emerson & Cuming, Stycast 2651, Westerlo, Belgium) to ensure the applicator was watertight. The rotation of the applicator is controlled remotely with negligible angular loss using an 80-cm long, 10-mm outer diameter (OD), flexible metallic shaft. A biocompatible envelope (1.3 mm thick) covers this metallic shaft. This envelope conforms to medical device safety requirements USP VI (U.S. Pharmacopoeia, class VI). Connections for the transducer supply are done with a PVC casing attached on the opposite end of the applicator. This casing is mounted on an UR 100 microcontrol unit (Microcontrol, Evry, France) to monitor the rotation of the applicator. Electrical connections were realised via a miniaturised 50-ohms coaxial cable that was 80-cm long with a 0.9-mm OD. A tube (1-mm OD) over the whole length of the applicator is for holding a guide wire previously introduced in the esophagus. The active part of the applicator was covered with a 65-μm thick latex balloon attached using watertight seals. This envelope attenuated the ultrasound pressure by about 9% and was inflated with degassed water to provide acoustic coupling between the transducer and the tissues. This envelope can be inflated via a syringe connected to the tank. During treatments, the transducer was cooled using a continuous flow of degassed water at room temperature (25°C) at a rate of 0.2 L/min. A peristaltic Masterflex pump (L/S model 7518-60, Cole-Parmer Instruments Co., Chicago, IL, USA) drove the water around a closed cooling circuit and through a 1-liter watertight tank of degassed water at room temperature. The treatment is monitored using an ultrasound imaging probe. A tube inside the applicator provides path for an UM2R ultrasound imaging probe (Olympus, Japan) which goes through the therapy applicator and perform a visualisation of the tissues in front of the therapy transducer (Fig. 2). The ultrasound imaging probe operates at a frequency of 12 MHz. In order to avoided damages to the imaging probe, it slides inside the applicator during high intensity ultrasound exposures.

The electrical power was delivered in continuous mode by a Kalmus model 150 CF power amplifier (Engineering International, Woodinville, WA, USA) driven by a Hameg HM8131 sinusoidal wave generator (Hameg, Frankfurt, Germany). Directional power meter (wattmeter/reflectometer (Rohde & Schwarz, Munich, Germany) fitted with a bidirectional coupling device probe (NAP Z7, Rohde & Schwarz, Munich, Germany) were inserted into the line between the amplifier output and the ultrasound applicator to determine the incident and reflected electrical power. The generator was controlled by a timer, which enabled emissions to be triggered manually and cut off automatically at the end of the desired exposure time. A radiation force balance, to the design of Hill [13], was used to measure the total acoustic power output of the ultrasound beam in degassed water at 21°C. An electro-acoustic efficiency of 61% at 10.4 MHz was measured.

### Patients

Previous animal experiments [11,12] led to an open pilot trial which was conducted between Sept, 2002 and Nov, 2004, after institutional review board approval from INSERM, Paris. Informed consent for the study was obtained from all patients and conformed to world medical association declaration of Helsinki Ethical Principles for Medical Research Involving Human Subjects, 2004. Criteria for inclusion were the presence of a histologically proven cancer of the esophagus, of any T, N and M stage, without invasion of the tracheo-bronchial tract on CT-scan or endoscopic sonography (EUS), considered non operable as a consequence of local or distant extent or of associated comorbidities. Patients with tracheo-bronchial invasion, tracheo-esophageal fistula, patients with an American Society of Anesthesiology (ASA) grade > 3 or Karnofsky index < 50%, patients with major coagulation impairment or bearing an esophageal stent were excluded. All procedures were undertaken under general anesthesia with airway intubation, as most endoscopic therapeutic procedures on the esophagus and also because it was a pilot study with relatively lengthy procedures. Pain assessment was done as usual in the post-interventional ward with opioid titration if necessary. It was planned to include 5 patients in this preliminary trial. The study endpoints were the feasibility of the treatment (with emphasis on the tumor visualization and position control during treatment) and the presence of effective tumor destruction in accordance with the visible tumor localization. Improvement in comfort (dysphagia visual scale graded 0–5) and quality of food intake, as well as undesirable side effects were also noted as secondary endpoints. The treatment was planned as a one session therapy, with a possibility of repeated sessions in case of initial positive but partial result.

### Ultrasound treatments

**Figure 3:**
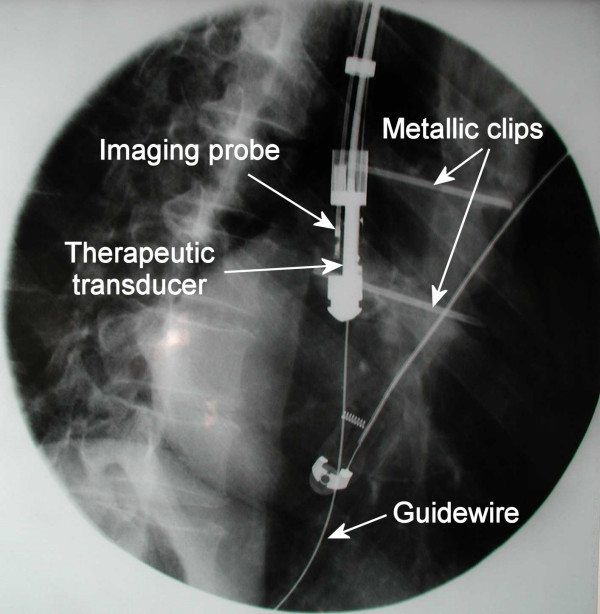
**Gross positioning of the therapeutic ultrasound applicator**. The therapeutic ultrasound applicator was placed in the esophagus under fluoroscopy after positioning metallic clips on the patient's skin.

**Figure 4:**
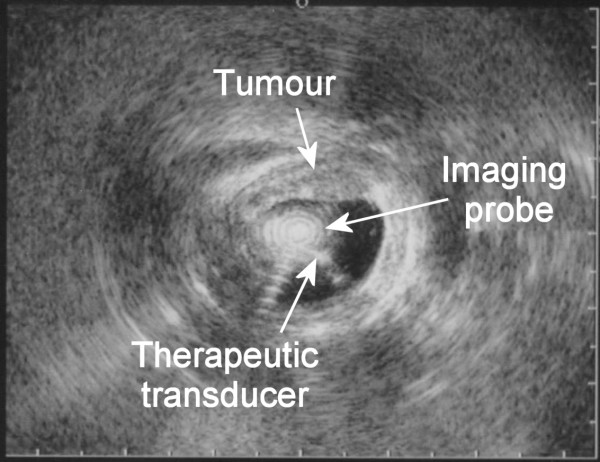
Sonogram of an esophageal tumor obtained during treatment.

Treatment was performed with the rotating transducer described above guided on a 0.035" guidewire, after bougienage when necessary (9 or 11 mm bougie). Gross positioning of the transducer was achieved under fluoroscopy after lipiodol injection in the upper and lower ends of the tumor as assessed by endoscopy, and positioning of metallic clips on the patient's skin (Fig. 3). Fine positioning and sectorial imaging, prior to and during treatment was achieved by use of the 12 MHz ultrasound imaging probe introduced in the dedicated channel of the therapeutic ultrasound applicator (Fig. 4). After confirming that the applicator was in correct position, the acoustic intensity at the surface of the transducer was adjusted (12 to 14 W/cm^2^) according to the thickness of the tumor. The duration of each exposure was 10 seconds. After each single lesion, the device was rotated 18 degrees around its axis, this motion being controlled manually from the external end of the device by means of a micrometer screw. These exposure conditions come from preliminary studies [11]. High intensity US application was circumferential or sectorial depending on EUS miniprobe and endoscopic appearance. After completion of a circumferential (20 single lesions) or sectorial application, the ultrasound applicator was moved within the stricture under fluoroscopy so as to create overlapping treatment rings. These maneuvers were repeated until the entire length of the stricture was treated.

### Follow-up

Follow-up was based on esophagoscopy with biopsies 8 days, 1 month and 3 months after therapy. Patients included in radiochemotherapy protocols underwent otherwise usual follow-up. Five patients have been included but only 4 have been treated with ultrasound. One patient included with a tumor of the esophagus at the level of the oro-pharyngeal junction was found to have a tracheo-esophageal fistula and was excluded. The other 4 patients are described in the results section and in table 1.

**Table 1:** Characteristics of individual patients.

No.	Gender	Age	Tumor type	TNM stage	Stricture C/L/T	Number of US sessions	Other treatments	Dysphagia Improvement	M1 status	M3 status	Survival
		
1	M	80	SCC	T3N1M0	360°/30/6 180°/15/6	1	5FU/CDDP	5 > 1	PR Clin: good Obstructive nodule Stent require	No tumor progression Clin: good No dysphagia	>4 mo
2	M	55	SCC	T1N0	110°/10/7 360°/9/9	3	5FU/CDDP	3 > 0	CR, proximal tumorPR, distal tumorClin: goodNo dysphagia	PRClin: poor, deteriorating general conditionRecurrent dysphagia	6 mo
3	M	83	AC	T3N2M1	360°/30/12	1	5FU	5 > 2			3 wk
4	F	86	SCC	T3NxM0	360°/20/7–12	1		5 > 2	PRClin: poorWeight loss (2 kg)		3 mo

*SCC*, squamous cell carcinoma; *AC*, adenocarcinoma; *TNM*, tumor, node, metastasis; *C/L/T*, circumference/length/thickness of tumor (mm); *5FU*, fluorouracil; *CDDP*, cisplatinum; *M1, M3*, 1 and 3 months after treatment; *clin*, clinical status; *PR*, partial response; *CR*, complete response; *US*, ultrasound.

## Results

Three patients (patients 1, 2 and 4) had squamous cell carcinoma (SCC) and one patient (patient 3) had adenocarcinoma (AC). In one case we found a double localization of a locally advanced SCC. Tumors were completely circumferential in 4 cases and sector-based (110° and 180°) in two cases. One patient (patient 1) was found to have a SCC of the median 1/3 of the oesophagus extending over 45 mm in length; the stricture was circumferential over the distal 30 mm and hemi-circumferential over the proximal 15 mm. Treatment was feasible in all patients, the main limitation encountered was the quality of treatment guidance using EUS miniprobe and the quality of the therapeutic applicator rotation. Tumor imaging was suboptimal by EUS miniprobe due to artifacts by multiple reflections on the therapeutic transducer and the presence of the balloon. In these cases, the contrast between tumors and normal tissues was less obvious when the miniprobe was inserted inside the therapeutic applicator. Additionally, the rotation of the applicator was not fully satisfactory, with fits and starts instead of a smooth rotation, in 2 out of the 4 patients treated. Therefore, depending of the quality of the probe rotation, the number of ultrasound exposures was adjusted during the procedure according to probe positioning visualized on ultrasound images.

Before the treatment, probe positioning in the stricture using fluoroscopy and EUS miniprobe was accurate and satisfactory in all cases. On average 25 ultrasound exposures were performed to treat circumferential tumors, 8 and 12 ultrasound exposures were performed for the 110° and 180° tumors respectively. In three cases (patients 1, 3 and 4) treatment was applied on several levels (two to three) depending on tumor length. The mean specific procedure duration, including set-up of equipment and US application was 37 ± 10 minutes (range 20 – 51 minutes). Objective tumor response was obtained in all cases. All patients recovered uneventfully and dysphagia improved significantly within 15 days, allowing for resuming a solid diet in three cases. Overall results are summarized in table 2.

**Table 2:** Summary of treatment results

		1 week	1 month	3 months
Patient 1	Biopsies	Positive in obstructive nodule	Positive in obstructive nodule	Positive in obstructive nodule
	Stricture reduction	Obstruction: 10%	Obstructive nodule at the upper part of the tumor	Obstructive nodule at the upper part of the tumor
	Endoscopic aspect	Necrosis on the whole length of the tumor	Necrosis on 75% of the tumor	No tumor progression

Patient 2 After the 3rd session of ultrasound	Biopsies	Positive in non necrotic areas	Positive in non necrotic areas	Not done
	Stricture reduction	No obstruction	No obstruction	
	Endoscopic aspect	Proximal tumor: Complete necrosisDistal tumor: 90% necrosed	Proximal tumor: No stenosis, complete necrosisDistal tumor: Infiltrating non-stenotic aspect	

Patient 3	Biopsies	Control endoscopy was contra-indicatedNo dysphagia		
	Stricture reduction			
	Endoscopic aspect			

Patient 4	Biopsies	Not documented	Not documented	Negative
	Stricture reduction	Obstruction: 90% → 50% Length: 2 cm → 1 cm	Obstruction: 50% Length: 1 cm	Obstruction: 50% → 70% Length: 1 cm
	Endoscopic aspect	Large necrotic area on the whole length of the tumor	Large necrotic area on the whole length of the tumor	Large necrotic area on the whole length of the tumor

### Patient 1

**Figure 5:**
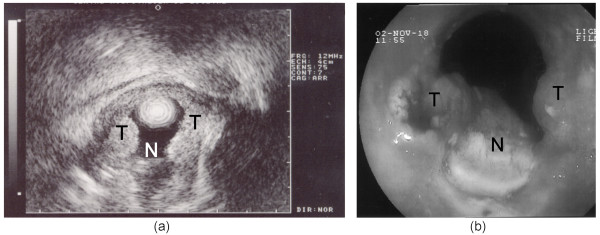
**Patient 1**. Tumor necrosis was observed on the whole length of the tumor, except at the proximal part where necrosis appeared to be incomplete. (a) Control endoscopy 8 days after the treatment. The necrosis was pushed distally with the endoscope. T, remaining tumor. N, Tumor necrosis. (b) EUS examination 8 days after the treatment.

An 80-year old man presenting with complete dysphagia was found to have a squamous cell carcinoma (SCC) of the median 1/3 of the oesophagus extending over 45 mm in length; the stricture was circumferential over the distal 30 mm and hemi-circumferential over the proximal 15 mm. The estimated tumor transversal diameter was 30 mm on CT-scan and EUS, and the TNM stage was usT3N1M0. Severe comorbidities (vascular and pulmonary) were found. Upper endoscopy revealed an associated adenocarcinoma of the antrum, which was classified as T1N0. Ultrasound was applied on 2 levels over 360° and on the proximal level over 180°, under EUS miniprobe control. The patient recovered uneventfully. Eight days after the treatment, tumor necrosis was observed on the whole length of the tumor, except at the proximal part where necrosis appeared to be incomplete. A second focus of incomplete necrosis was noted on 5 mm in the centre of the tumor. The necrosis was pushed distally with the endoscope and the stricture was subsequently passed easily without need for further dilation (Fig. 5). Dysphagia improved considerably during the following 2 weeks, allowing for solid food intake, but recurred at 4 weeks: control upper endoscopy at 1 month showed an obstructive nodule at the upper part of the tumor, while the more distal part remained persistently patent. Despite a partial efficacy of the US therapy, a second session was declined and radiochemotherapy was started, along with the insertion of a self-expandable covered stent. Control endoscopy at four months showed no tumor progression and the patient presented no dysphagia, with a patent stent.

### Patient 2

**Figure 6:**
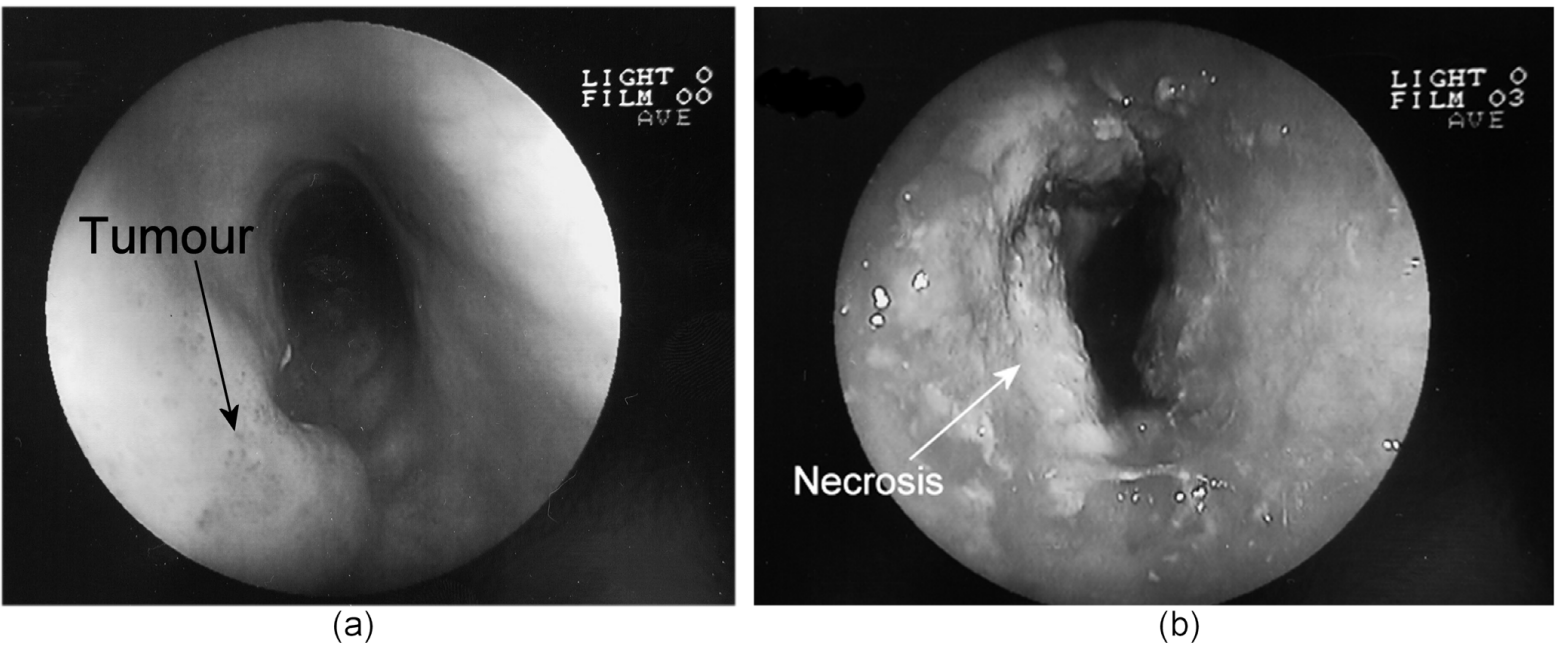
**Patient 2**. Control endoscopy of the esophageal tumor. (a) before and (b) 10 days after the treatment.

A 55 year-old man had previously undergone radiochemotherapy (5FU-CDDP-based) for a double localization of a locally advanced SCC of the upper and median 1/3 of the oesophagus. A complete response had been established in 2002. One year later, follow-up endoscopy revealed recurrences on both initial sites, without significant symptoms. On EUS, the upper tumor was graded T1N0 and the lower tumor appeared to infiltrate the muscularis propria, without evidence of lymph node involvement. Both tumors did not exceed 4 mm in thickness. It was decided to attempt ultrasound therapy. A first treatment session was undertaken with decreased power output in order to prevent transmural necrosis and possible fistula. At control endoscopy at 8 days, there was no evidence of tissue ablation and biopsies showed persistence of carcinoma cells. A second treatment session was subsequently proposed, with the same treatment parameters. The control endoscopy 10 days after this second treatment demonstrated non significant change and still positive biopsies. Three months later, the patient presented with mild dysphagia and a moderate weight loss. On the patient's request, a 3rd treatment session was decided; since the tumor thickness had increased in the meantime to reach a maximum of 7 mm, usual treatment parameters were applied. Recovery was uneventful, a mild cervical pain requiring 2 g paracetamol IBD during 3 days was noted, and dysphagia disappeared completely within 10 days, along with an improvement in the patient's performance status. Control endoscopy at 10 days showed a complete necrosis of the proximal lesion (Fig. 6) and an estimated 90% necrosis of the more distal one were achieved after three ultrasound treatment sessions. At 1 month, the patient maintained the same performance status (Karnofsky 90%), had a steady weight and no dysphagia. Endoscopy showed no stenosis, an infiltrating, non-stenotic aspect of the distal lesion and a nearly complete regression of the proximal lesion. Two months later (3 months after the third ultrasound session), the patient presented with recurrent dysphagia and deteriorating general condition. He was admitted in a primary referral centre, where no endoscopy was done, and a complementary radiotherapy was discussed. During this hospital stay, a sepsis developed with a septic shock and coma. Blood cultures and cephalo-rachidian fluid were positive for E. coli sp., leading to the diagnosis of meningo-encephalitis secondary to a septicaemia of unknown origin.

### Patient 3

An 83 year-old man presenting with dysphagia and a major weight loss (-15 kg) was diagnosed to have an adenocarcinoma of the distal oesophagus developed on Barrett's oesophagus. The tumor was staged usT3N1 on EUS and N2M1 on CT-scan (cervical lymph nodes and multiple liver metastases). The oesophageal stricture was measured at 30 mm in length and 12 mm in thickness. Additionally, the patient presented a stenosis of benign appearance of the upper 1/3 of the oesophagus. Although no specific cause was demonstrated to explain this second stenosis, a mediastinal tumor extent was suspected. In view of the poor prognosis, the strategy included an endoscopic stenting of the upper oesophageal stenosis, an oral chemotherapy based on oral 5FU and an ultrasound therapy of the lower oesophageal stricture with the aim of comfort improvement. The ultrasound treatment was performed on 2 levels of the ultrasound transducer with a satisfactory visualization of the tumor and an oesophageal stent was implanted. The patient recovered uneventfully and his dysphagia improved significantly within 10 days, allowing for resuming a solid diet. However, the performance status decreased continuously with the progressive development of jaundice due to the hepatic metastases. Subsequently, the control endoscopy was contra-indicated and the patient died 3 weeks after ultrasound therapy.

### Patient 4

An 86 year-old woman presenting with severe dysphagia and a weight loss of 14kg was found to have an oesophageal SCC of the lower 1/3 of the oesophagus extending over 20 mm in length and 7 to 12 mm in thickness (25 mm in overall diameter). The tumor was staged usT3Nx on EUS and M0 on CT-scan. Due to her old age, oesophagectomy was not a reasonable option and US was proposed for palliation as the sole therapeutic method. The treatment was achieved over 2 levels of the US transducer. The patient recovered uneventfully. After 8 days, the dysphagia score regressed from 5 to 2 and the follow-up endoscopy demonstrated a large necrotic area on the whole length of the tumor, predominantly in the centre of the lesion, and a regression of the percentage of oesophageal obstruction from 90% to an estimated 50%, with a stricture length reduced from 2 to 1 cm. At 1 month, the dysphagia score was identical, the patient had lost another 2 kg, and the endoscopic observations were unchanged as compared to the first follow-up endoscopy. After 3 months, the patient's general condition remained fair, but she had lost another 2 kg, her dysphagia had increased from a score of 2 to 3, allowing only a soft diet and the endoscopy showed an increase in the degree of the stricture estimated at 70% without any increase in length (10 mm as previously). Biopsies were negative. Bougienage was done up to 13 mm without further stenting.

## Discussion

The efficacy of local tumor destruction by high-intensity ultrasound therapy has been has been established for decades, both experimentally and clinically [14,4,3]. Intraluminal US therapy with a plane rotating transducer is a new application that is particularly suitable for the tumors of the digestive tracts. In addition, the rotation of the transducer allows for treatment of specific segments of the circumference of a hollow organ, which makes the method particularly suitable for noncircumferential esophageal tumors.

Prior to clinical studies, encouraging experimental results have been achieved with intraluminal flat transducers in pigs which is an ideal animal to study the different treatment options available for the treatment of esophageal tumor, in view of its size and physiology similar to humans. However, there is no established tumor model in pigs due to the absence of porcine liver tumor cell lines which can be used to create esophageal tumors in the pig. Nevertheless, the efficacy of HIFU on tumors as been demonstrated with many clinical studies and is not dependent of the soft tissues that are sonicated [14]. In addition, the experimental histological studies have shown that the lesions created in pigs were homogeneous [11] and our experimental results in pigs were considered to be applicable to humans. The present clinical series provides further evidence of the feasibility and patient tolerance of the method. The US treatment was well tolerated by all patients.

The intraluminal US applicator was developed for direct insertion in the esophagus and over a 0.035-inch guidewire. The applicator was in all cases easily exchanged over the guidewire and its insertion within the esophagus was also feasible in all patients, as was transducer positioning under fluoroscopic guidance. The system and techniques developed free the endoscopist's hands for the purpose of controlling the actual application of the US energy.

The clinical pilot trial was planned to be continued if the first results had been enough satisfactory. We observed several limitations, related in particular to the rotation of the applicator. Both of these 2 criteria were found unsatisfactory in 2 out of the 4 patients actually treated. This is explained by a reduced image quality of the US miniprobe when introduced into the applicator, which is worsened by the presence of the balloon. This is responsible for impairment in tumor imaging and subsequent transversal positioning in case of sectorial tumor infiltration. The uneven rotation of the probe was due to frictions over the applicator induced by the presence of tumor tissue, thus precluding a smooth and accurate transducer rotation. None of these factors could have been foreseen from preclinical studies, since there was no animal model of esophageal tumor available. Excess deep coagulation necrosis is expected to potentially induce mediastinal perforation. This complication was not observed in this pilot study, despite inadequate imaging of the tumor and surroundings in two cases. Indeed, a complete in-depth destruction of tumors infiltrating the muscularis propria might create esophageal perforation and mediatinal or oeso-tracheal fistulae, depending on tumor location. However, this hazard is probably less problematic compared with destruction methods such as radiofrequency or bipolar electrosurgery in which coagulation is both less rapid and less accurate. Ultrasound ablation induces a progressive necrosis which allows enough time for inflammation and sclerosis to develop and form an inflammatory barrier against sepsis. This has been previously demonstrated in preliminary short-term animal experiments [11].

Patients with radiochemotherapy treatments were included in this study. Nevertheless, confusion between radiation and ultrasound-induced necrosis was unlikely since patients did not undergo radiotherapy during the same period as ultrasound. In patients receiving chemotherapy, tumor response does not translate into brutal necrosis, but rather into progressive tissular thaw. Additionally, an early endoscopic control was done 8 days after ultrasound exposure has clearly demonstrated the sharply delineated US-induced necrosis.

Nevertheless, this clinical study has shown that the treatment was feasible and induced tumor necrosis as well as a significant and relatively rapid symptomatic improvement in these severely ill patients. We have decided to resume these clinical studies as soon as the above mentioned problems will be appropriately addressed. Our main goals are now to develop multitransducer applicators which can be operated without any mechanical rotation, once positioned longitudinally, and to prefer magnetic resonance imaging to ultrasound diagnostic to achieve tumor imaging [15]. Additionally, long-term follow-up studies are needed to assess late treatment results in patients who have small tumors without evidence of regional or distant extension.

Future studies will need to determine whether US application is only a new palliative method, perhaps abolishing the need for stents in some cases, or whether it can also be curative in selected high-risk patients or those with localized tumors. Ultrasound therapy can be discussed in a neo-adjuvant setting, in order to reduce the tumor bulk and reinforce the tumor response before surgery, as a complement to RT-CT. However, one should be aware of the potentially deleterious mediastinal inflammation which might make resection difficult or impossible. Finally, the indications most likely to be of interest in esophageal cancer are listed as follows:

-superficial tumors with important lateral spread (longitudinal and circumferential) precluding endoscopic resection by current methods (endoscopic mucosal resection and endoscopic submucosal dissection).

-palliation in locally advanced or metastatic tumors for desobstruction and as a complement to chemotherapy.

-in combination with or as an alternative to RT-CT in cases of recurrence after surgery or RT-CT, resistance to RT-CT or failed resection (tumor not reseceted).

## List of abbreviations

US: Ultrasound; EUS: Endoscopic sonography; ASA: America, Society of Anesthesiology classification; SCC: squamous cell carcinoma; AC: adenocarcinoma; OD: outer diameter; CT-scan: computerised tomography scan; RT-CT: Radiochemotherapy.

## Competing interests

The authors declare that they have no competing interests.

## Authors' contributions

DM participated in the design of the ultrasound device, participated in the conception and the coordination of the study and drafted the manuscript. FP participated in the conception and the coordination of the study and drafted the manuscript. JF participated in coordination of the study. YT participated in the design of the ultrasound device and built the ultrasound device. DC participated in the design of the ultrasound device and in the conception and the coordination of the study. All authors read and approved the final manuscript.
